# Control of the thymic medulla and its influence on αβT‐cell development

**DOI:** 10.1111/imr.12406

**Published:** 2016-04-18

**Authors:** Beth Lucas, Nicholas I. McCarthy, Song Baik, Emilie Cosway, Kieran D. James, Sonia M. Parnell, Andrea J. White, William E. Jenkinson, Graham Anderson

**Affiliations:** ^1^ MRC Centre for Immune RegulationInstitute for Immunology and ImmunotherapyMedical SchoolUniversity of BirminghamBirminghamUK

**Keywords:** thymus, thymocyte, thymic epithelium, tolerance

## Abstract

The thymus is a primary lymphoid tissue that supports the generation of αβT cells. In this review, we describe the processes that give rise to the thymus medulla, a site that nurtures self‐tolerant T‐cell generation following positive selection events that take place in the cortex. To summarize the developmental pathways that generate medullary thymic epithelial cells (mTEC) from their immature progenitors, we describe work on both the initial emergence of the medulla during embryogenesis, and the maintenance of the medulla during postnatal stages. We also investigate the varying roles that receptors belonging to the tumor necrosis factor receptor superfamily have on thymus medulla development and formation, and highlight the impact that T‐cell development has on thymus medulla formation. Finally, we examine the evidence that the thymic medulla plays an important role during the intrathymic generation of distinct αβT‐cell subtypes. Collectively, these studies provide new insight into the development and functional importance of medullary microenvironments during self‐tolerant T‐cell production in the thymus.

This article is part of a series of reviews covering The Thymus, Lymph nodes, and Lymphatics appearing in Volume 271 of *Immunological Reviews*.

## Introduction

T cells expressing the αβ form of the T‐cell receptor (αβTCR) are generated in the thymus from migrant lymphoid progenitors that arise in the liver during embryonic life, and then in the bone marrow at postnatal periods 1, 2. As the thymus contains no intrinsic ability to generate haemopoietic stem cells, it must be regularly seeded by migrant T‐cell progenitors in order to support the continued intrathymic production of naive T cells 3, 4. The importance of the thymus for normal immune system function is clear from studies that document the direct impact of genetic alterations on thymic development, and the effect this has on T‐cell production. For example, in both rodents and humans with naturally occurring mutations in the Foxn1 gene, thymus tissue fails to form correctly during embryonic life, which results in a loss of T cell‐mediated immunity 5, 6, 7, 8. Similarly, there are indications that in man, the surgical removal of normal thymic tissue may also impact T‐cell production and immune system function later in life 9, 10.

Given the importance of the thymus, intrathymic T‐cell development is a well‐studied process. While the precise nature of thymus colonizing cells is not clear, their downstream descendants, early thymus progenitors, are defined by a CD4^−^ CD8^−^ CD44^+^ CD25^−^ CD117^+^ phenotype 11, 12. Subsequent stages of differentiation within CD4^−^ CD8^−^ stages include CD44^+^ CD25^−^ double negative 1 (DN1) which can be further subdivided on the basis of CD24 and CD117, and then CD44^+^ CD25^+^ DN2, CD44^−^ CD25^+^ DN3 13. Successful rearrangement and expression of TCRβ proteins as part of the pre‐TCR complex triggers thymocyte differentiation and expansion via an intermediate CD44^−^ CD25^−^ DN4 stage, resulting in the emergence of a large pool of CD4^+^ CD8^+^ thymocytes expressing low levels of αβTCR that reside within the thymic cortex. Such cells represent the progenitors of CD4^+^ 8^−^ and CD4^−^ 8^+^ thymocytes that represent the most mature stage of T‐cell development in the thymus 14, and enter the peripheral T‐cell pool as recent thymus emigrants (RTE) 15.

Given the complexity of intrathymic T‐cell development, it is perhaps not surprising that thymocytes are unable to control their own maturational program. Instead, they must continually receive signals from their surrounding thymic microenvironments to ensure that their step‐wise development takes place correctly 16. During postnatal stages, lymphoid progenitors that enter the thymus via blood vessels at the corticomedullary junction migrate outwards through the cortex, resulting in the accumulation of CD25^+^ CD44^−^ DN3 progenitors within the subcapsular region. During these early phases of development, thymocytes remain in contact with cortical thymic epithelial cells (cTEC) that form a complex reticular network of stromal cell support throughout the cortex. Several studies have shown that interactions with cTEC are important during both early and later stages of thymocyte maturation. For example, cTEC express the Notch ligand Delta‐like 4, which drives the formation of large numbers of cortical CD4^+^ CD8^+^ thymocytes 17, 18, 19. To allow further differentiation, cTEC mediate thymocyte positive selection by presenting a specialized array of self‐peptides via major histocompatibility complex (MHC) class I and class II molecules 20, so that cells capable of αβTCR‐MHC recognition are rescued from apoptosis. Importantly, positive selection is essential for newly selected thymocytes to gain entry into the thymic medulla 2, an intrathymic microenvironment that is anatomically and functionally distinct from the cortex. By acting as a reservoir of newly selected thymocytes and as a site for T‐cell tolerance induction, the thymus medulla shapes antigen recognition in the naïve T‐cell pool by limiting the development of cells that recognize self‐antigens, thereby limiting the chance of autoimmunity 21. Collectively, these studies underline the complexity of thymocyte development, and emphasize the importance of the cortical and medullary areas that provide stromal cell support during this process. Elsewhere in this volume, Takahama *et al*. review the development and function of the thymic cortex. Here, we focus on the thymic medulla, beginning with studies that facilitated its analysis, and then summarizing current knowledge of its developmental origins and functional importance.

## Sorting out stroma: a brief history

Early studies that combined the transplantation of embryonic thymus and bone marrow between wildtype (WT) and nude mice were among the first to demonstrate the link between epithelial cell development and thymus function 22. In order to gain insight into the role of TEC during T‐cell development, early studies aimed to isolate and propagate these cells using *in vitro* monolayer cultures that often required the presence of fibroblast feeder layers. However, due to the limited isolation techniques available at the time, TEC heterogeneity remained poorly defined and often relied upon morphological analysis. As a consequence, the ability to recapitulate and study thymocyte development *in vitro* in the presence of defined thymic stromal cells was lacking 23, 24, 25. As isolation methods improved, TEC heterogeneity could be revealed by distinct patterns of cytokeratin expression 26, 27, 28, although this still did not enable the isolation and study of distinct TEC subsets. Such studies only became possible through the availability of reagents that recognized cell surface determinants on TEC, and that could be used in either magnetic bead or fluorescence activated cell sorting‐based sorting protocols. These include the fucose‐binding lectins tetragonolobus purpureas agglutinin and ulex europeus agglutinin (UEA) 29, the latter still widely being used to identify and isolate mTEC. In addition, the generation of multiple monoclonal antibodies has greatly aided in TEC isolation, including clone G8.8 that recognizes the pan‐epithelial determinant EpCAM1 30, the mouse thymus stroma antibody series 31, and NLDC‐145 32 and 6C3 33 that identify CD205 and Ly51 expressed by cTEC. The availability of these reagents have helped to establish ‘Standard Operating Procedures’ that are widely used in the isolation of EpCAM1^+^ UEA^+^ mTEC that are Ly51^−^/CD205^−^, and EpCAM1^+^ UEA^−^ cTEC that are CD205^+^ or Ly51^+^.

While methods for the isolation of TEC subsets improved, *in vitro* systems were still limited in their ability to support T‐cell development, which perhaps could be explained at least in part by the loss of expression of Foxn1 and Dll4 by TEC grown in two‐dimensional monolayer cultures 34, 35. Consequently, we aimed to establish new culture techniques that supported the functional analysis of purified thymic stromal cell types *in vitro*. As three‐dimensional cultures had been successfully used in the analysis of other epithelial tissues 36, we established a system based on fetal thymus organ culture (FTOC) 37, 38, 39, 40 that enabled the re‐association of defined thymic stromal populations within three‐dimensional *in vitro* cultures. In initial studies using reaggregate thymus organ cultures (RTOC), we showed that positive selection of a single cohort of CD4^+^ CD8^+^ thymocytes could be analyzed and manipulated 41, 42, 43, 44. Furthermore, by varying the developmental stage of T‐cell precursors used to form RTOC, stage‐specific requirements for distinct thymic stromal populations were identified for the first time. For example, while TEC alone were both essential and sufficient for the maturation of CD4^+^ CD8^+^ thymocytes, a combination of TEC and mesenchyme cells was shown to be required for CD4^−^ CD8^−^ T‐cell precursor development 45, 46. Importantly, FTOC and RTOC that have formed *in vitro* can be transplanted under the kidney capsule of recipient mice, providing a powerful approach to combine *in vitro* manipulation with the study of TEC populations *in vivo*. With regard to the thymic medulla, and as described in further detail in subsequent sections, these techniques have been particularly important in identifying and tracking the developmental potential of defined mTEC progenitor populations 47, 48, 49, 50, and demonstrating the importance of mTEC during the induction of T‐cell tolerance mechanisms *in vivo* 51, 52, 53, 54.

## Pathways in mTEC development

### mTEC progenitors

**Figure 1:**
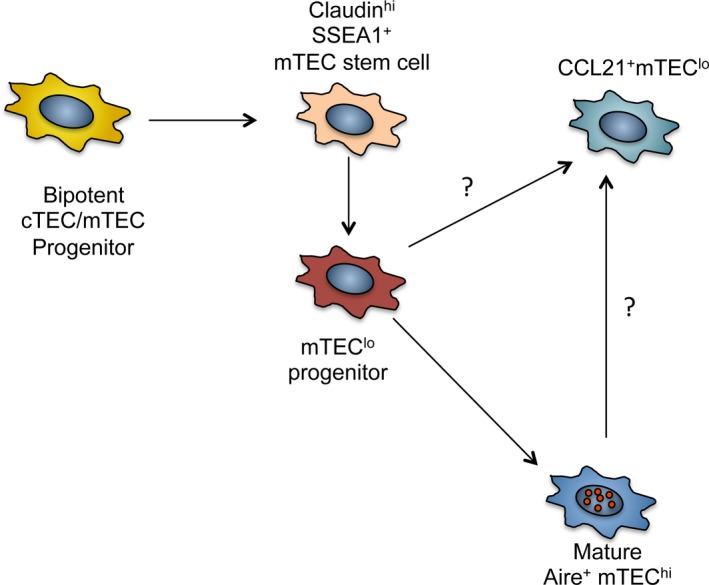
**Pathways in medullary thymic epithelial cells (m** **TEC** **) development.** The identification of SSEA1^+^ mTEC stem cells downstream of bipotent TEC progenitors marks an important stage in thymus medulla development. Such cells have the potential for both self‐renewal and the generation of mature mTEC progeny that establish thymic tolerance. The mTEC ^lo^ compartment, defined by low levels of MHC class II and CD80, are known to contain the RANK ^+^ progenitors of Aire^+^ mTEC ^hi^. However, the frequency of these progenitors within the mTEC ^lo^ compartment, and their detailed phenotypic properties, are not known. Additionally, the description of LTβR‐dependent CCL21^+^ mTEC ^lo^ suggests that not all mTEC ^lo^ may be the progenitors of mTEC ^hi^, suggesting developmental heterogeneity within the mTEC ^lo^ compartment.

Consistent with their common endodermal germ layer origin 55, functional assays showed that the cTEC and mTEC lineages both arise from bipotent TEC progenitors 48, 56. Importantly, bipotent progenitors have been recently isolated and further defined using a combination of markers (e.g. α6 integrin, Sca1, CD24) and assays, which will aid in understanding both cTEC and mTEC development 57, 58, 59, 60. Indeed, the events that occur downstream of bipotent progenitors, notably in relation to development of the mTEC lineage, have been the focus of investigation for many studies in recent years. While mTEC heterogeneity has been well reported, the first direct evidence of mTEC progenitors was provided at a functional, rather than phenotypic, level. Notably, the demonstration that individual islets of mTEC could be derived from a single cell, provided the starting point to identify and study TEC progenitors that are committed to the mTEC lineage 47. In our own studies 51, and those of others 61, by monitoring the developmental potential of purified TEC populations using *in vitro* RTOC, we showed that MHC class II^low^ CD80^−^ mTEC (mTEC^low^) could generate MHC class II^high^ CD80^hi^ (mTEC^hi^), providing an indication of one of the precursor–product relationships within the mTEC lineage. Since then, and using similar experimental approaches, mTEC progenitors – including those that give rise to mature Aire^+^ mTEC – have been further defined. For example, Hamazaki *et al*. 50 have shown that a subset of TEC with the potential to generate mTEC but not cTEC could be defined by expression of Claudin‐3 and ‐4 (Cld3/4). Furthermore, an SSEA1^+^ subset of Cld3/4^+^ cells was recently reported to possess both self‐renewing capabilities and the long‐term potential to generate mTEC *in vivo* 49. The identification of such SSEA1^+^ mTEC stem cells (mTECSC) is important as it provides an opportunity to study stages in TEC development that occur immediately downstream of bipotent TEC progenitors (*Fig.* 1). In addition, and consistent with the presence of mTECSC, Ohigashi *et al*. 62 recently showed that in the adult thymus, mTEC‐restricted progenitors that are generated downstream of bipotent progenitors during embryonic life are responsible for maintenance of the mTEC compartment in the steady state and its regeneration following injury. Interestingly, an earlier study showed that TEC with mTEC features could be identified in the absence of Foxn1 expression 63, suggesting that the initial emergence of the mTEC lineage can occur in the absence of this key transcription factor. Whether such Foxn1‐independent mTEC progenitors arise from SSEA1^+^ mTECSC, or represent an alternative pathway of mTEC lineage development, is not clear.

To directly examine the lineage relationships of cTEC and mTEC, we screened developing populations in the mouse embryonic thymus using a panel of markers, including CD40 and the cTEC marker CD205 64. Using this approach, we saw a pattern of expression defined by the serial acquisition of first CD205, and then CD40. By E15 of gestation, we showed that while CD205^+^ CD40^−^ cells expressed cTEC‐related markers such as β5t and Cathepsin‐L, CD205^−^ CD40^+^ cells expressed a range of mTEC markers including Aire, Cathepsin‐S, and osteoprotegerin (OPG). Based on this evidence, we proposed that CD205^+^ CD40^−^ cells represented cTEC‐restricted progenitors 64. Surprisingly however, when we assessed the developmental potential of embryonic CD205^+^ CD40^−^ TEC *in vivo*, we found they gave rise to both the cTEC and mTEC lineages, including Aire^+^ cells 65. Furthermore, *in vitro* stimulation assays showed that a proportion of CD205^+^ CD40^−^ cells expressed RANK, a key regulator of the mTEC lineage 65. Taken together, such findings showed that at least in the embryonic thymus, mTEC could be derived from progenitors that are defined by the expression of markers of the cTEC lineage. Importantly, similar observations were reported in other studies involving either fate mapping of TEC development using β5tCre mice 66, or assessment of the developmental potential of cells expressing additional cTEC traits, including IL7^YFP^ and CCRL1 67, 68. Collectively, the evidence from these studies suggested a ‘serial progression model’ of embryonic TEC development 69, in which bipotent TEC progenitors initially acquire a cTEC‐like phenotype, which is then followed by the loss of cTEC markers and potential, resulting in the generation of mTEC. Given that mTEC represent a dynamic population that is continually replaced from a progenitor pool, it is important to note that studies have not yet been reported that directly address whether a similar developmental process continues to take place beyond the embryonic thymus and throughout postnatal life.

Importantly, although advances have been made in understanding pathways in mTEC development, the location of immature TEC progenitors within organized thymic tissue remains poorly understood. Recently, Onder *et al*. 70 used a variety of fate mapping approaches in mice to investigate the anatomical positioning of mTEC progenitors in relation to medulla formation. In their studies, they indicated that mTEC progenitors defined by expression of podoplanin were identifiable at the corticomedullary junction, making them well placed to contribute to continued generation of medullary areas. Interestingly, other studies have generated three‐dimensional reconstructions of the adult mouse thymus, and shown that the medulla consists of around 200 small areas 71. Whether each individual area is generated from a single mTEC progenitor residing at the corticomedullary junction is not clear. Moreover, given the identification of mTEC stem cells 49 and the heterogeneity in mTEC that is emerging from other studies 72, 73, it will be interesting to determine whether podoplanin^+^ progenitors represent a population that can give rise to all mature mTEC subsets in a sustained manner.

One of the key aspects of further defining stages in mTEC development has been the availability of tools and reagents that can be used to define known functional regulators of thymus medulla formation. For example, the generation of monoclonal antibodies to analyze patterns of Aire expression 74 has played an important part in identifying mTEC subsets of functional relevance. In contrast, expression patterns of other key mTEC regulators including RANK have been difficult to define. Given the paucity of suitable antibodies, we recently generated BAC transgenic mice in which expression of the fluorescent protein Venus can be used to monitor RANK expression. In initial analysis of the mTEC compartment of adult mice, we identified complex heterogeneity with regard to RANK expression. For example, both mTEC^lo^ and mTEC^hi^ compartments contained RANK‐Venus^+^ and RANK‐Venus^−^ subsets. Moreover, subdivision of mTEC^lo^ and mTEC^hi^ cells on the basis of expression of CCL21 and Aire showed that RANK‐Venus did not fully overlap with either marker. While such initial studies show that further work is required to understand the lineage relationships of RANK^+^ and RANK^−^ mTEC, they suggest that the subdivision of the mTEC lineage into mTEC^lo^ and mTEC^hi^ is over‐simplistic. Indeed, although mTEC^lo^ are known to contain the progenitors of mTEC^hi^ cells, it may be that mature cells also reside within the mTEC^lo^ compartment. Perhaps in support of this, a recent study 75 showed that a subset of mTEC^lo^ cells expresses the CCR7 ligand CCL21 in a lymphotoxin β receptor (LTβR)‐dependent manner. This study is important as it demonstrates heterogeneity within the mTEC^lo^ compartment, and indicates that at least some mTEC^lo^ cells express molecules that might functionally influence thymocyte development. Thus, it is still currently unclear what proportion of the mTEC^lo^ compartment represents the immature progenitors of mTEC^hi^, and whether additional mTEC^lo^ cells exist that represent mature cells in a sub‐branch of the mTEC lineage that does not include an mTEC^hi^ stage.

## The TNFR superfamily and the thymus medulla

The nuclear factor (NF)‐κB signaling pathway is known to be an important regulator of thymus medulla formation 76, 77. Signaling through various members of the tumor necrosis factor receptor superfamily (TNFRSF) activates this pathway and to date, four receptors (RANK, OPG – a decoy receptor for RANK, CD40, and LTβR) have been linked to mTEC differentiation. The requirement of NF‐κB activation for mTEC development is evident from mice deficient in RelB, a subunit of the NF‐κB complex. Indeed, Relb^−/−^ mice have a dramatically reduced medullary compartment, accompanied by a loss of UEA‐1^+^ mTEC 77, 78, 79. Importantly, in addition to severe multi‐organ autoimmunity 77, these mice have additional immune system abnormalities including reduced frequencies of thymic dendritic cells (DC) and a failure in lymph node organogenesis. Given the complex phenotype of Relb^−/−^ mice, it has been difficult to determine whether the autoimmunity seen in is due to a specific requirement for Relb expression by TEC for central tolerance. To address this issue, we employed a thymus transplantation system in which alymphoid 2‐deoxyguanosine (dGuo)‐treated Relb^−/−^ FTOC were transplanted into athymic nude mice, thereby compartmentalizing the RelB defect to TEC 52. In these experiments, nude mice that received Relb^−/−^ TEC grafts developed multiple symptoms of autoimmunity, including the presence of lymphocytic infiltrates in the liver and autoantibodies in the serum. Interestingly, WT hosts grafted with Relb^−/−^ stroma showed no signs of autoimmunity, presumably due to peripheral tolerance mechanisms involving Foxp3^+^ regulatory T cells (T‐Reg) generation by the host thymus 52. Such observations are in line with other studies that also highlighted the importance of NF‐κB signaling in medulla development for tolerance induction. For example, aly/aly mice deficient in NF‐κB inducing kinase (Nik) also show mTEC abnormalities and autoimmunity 53, 80. In addition, TRAF6‐deficient mice also have medullary abnormalities including a reduction in Aire^+^ mature mTEC. Similar to studies employing Relb^−/−^ thymus grafts, engraftment of embryonic TRAF6^−/−^ dGuo‐treated FTOC into nude mice resulted in autoimmunity characterized by multi‐organ immune infiltrates 81. In a more recent study, and again to specifically address the requirement of TRAF6 expression by TEC, Bonito *et al*. 82 generated Foxn1Cre × TRAF6 ^fl/fl^ mice. These mice were reported to have a diminished thymic medulla development, a reduced frequency of Aire^+^ mTEC and hallmark features of autoimmune hepatitis, including the presence of autoantibodies in the sera. Combined, these studies indicate the importance of NF‐κB signaling in the formation of thymic microenvironments for tolerance induction, and have initiated further studies to identify the cell surface receptors that employ this pathway during thymus medulla development.

### RANK‐mediated mTEC development

In addition to NF‐κB signaling, mTEC maturation is dependent on hematopoietic crosstalk, a process in which developing thymocytes provide differentiation signals necessary for the regulation of TEC development. Such crosstalk involves signaling through mTEC expression of TNF receptors and is a process that occurs in both the embryonic and adult thymus 83, 84, 85, 86, 87. In addition, and in contrast to previous reports 88, we showed that thymic microenvironments remain receptive to crosstalk in the prolonged absence of T‐cell development, arguing against the presence of a developmental window in embryonic development during which initial crosstalk must take place 89. In relation to the role of crosstalk during embryonic thymus development, studies from our own lab have revealed a role for embryonic RORγt^+^ innate lymphoid cells and progenitors of Vγ5^+^ dendritic epidermal T cells in the emergence of the first cohorts of Aire^+^ mTEC. For example, the addition of RANKL expressing innate lymphoid cells from embryonic spleen to TEC progenitors in RTOC triggered mTEC maturation, including the generation of CD80^+^ mTEC^hi^ cells expressing Aire 51. Likewise, similar experiments adding purified RANKL‐expressing Vγ5^+^ thymocytes to RTOC also induced Aire^+^ mTEC development, which fits well with their close proximity to mTEC *in vivo* 90. Interestingly, the generation of embryonic mice deficient in both RORγ expression and γδT cells showed a further reduction in Aire^+^ mTEC compared to mice deficient in either cell type alone 90, suggesting that during embryonic thymus development, innate lymphoid cells and DETC progenitors synergize to trigger mTEC development.

Consistent with the importance of RANKL^+^ cells during thymocyte–TEC crosstalk, studies using embryonic and adult mice have shown a clear requirement for RANK in mTEC maturation. For example, embryonic mice deficient in RANK or RANKL have a complete absence of Aire^+^ mTEC 51, 86. Further evidence of RANK signaling for mTEC differentiation was provided by experiments in which *in vitro* stimulation of dGuo‐treated FTOC with either recombinant RANKL or agonistic anti‐RANK antibody resulted in the upregulation of CD80 and Aire expression by mTEC 51, 91. In contrast to the embryonic thymus, adult mice deficient in RANK or RANKL have reduced, rather than absent, Aire^+^ mTEC. Interestingly, Akiyama *et al*. 86 observed Aire expression in the RANKL^−/−^ thymus at postnatal day 3, providing strong evidence that after birth, additional interactions could influence Aire^+^ mTEC development in the absence of RANK signaling. Finally, and consistent with the importance of the RANK–RANKL axis in thymus medulla development, we showed recently that a population of Aire^+^ mTEC^hi^ can be defined by expression of OPG, a soluble decoy receptor for RANKL 72. Moreover, mice deficient in OPG have a large thymic medulla and increased numbers of Aire^+^ and Aire^−^ mTEC^hi^ and mTEC^low^ cells, all of which are RANK^+^ targets of OPG‐mediated control 72, 85, 92. Thus, while OPG expression is limited to a particular subset of mTEC, it can operate via *cis* and *trans* mechanisms to regulate homeostasis within the global mTEC compartment.

### CD40–CD40L interactions

Several studies have also highlighted the importance of an additional TNFR, CD40, in regulation of the thymus medulla in the postnatal period 85, 86, 87, 93, 94. Interestingly, and in contrast to the requirement for RANK, CD40 signaling appears to have a more subtle effect on mTEC maturation. For example, adult mice doubly deficient in RANK and CD40 show a greater reduction in Aire^+^ mTEC compared to RANK deficiency alone, demonstrating the requirement for co‐operation between RANK and CD40 in postnatal mTEC differentiation 86. These experiments suggest an essential requirement for RANK signaling during the initial emergence of Aire^+^ mTEC in the embryo, whereas subsequent postnatal mTEC differentiation relies on co‐operation between both CD40–CD40L and RANK–RANKL signaling.

In the adult thymus, and again consistent with the importance of thymocyte crosstalk, mature thymocytes are the major source of RANKL and CD40L. RANKL is expressed preferentially by CD4^+^ CD8^−^ thymocytes and also by a small population of CD4^−^ CD8^+^ thymocytes, whereas CD40L is expressed exclusively by CD4^+^ CD8^−^ thymocytes 95. Studies using mice deficient in CD4^+^ CD8^−^ and CD4^−^ CD8^+^ thymocytes, either individually or combined, have investigated the importance of each cell type in mTEC maturation. While mice deficient in MHC class I expression and consequently CD4^−^ CD8^+^ thymocytes have unaltered numbers of mature Aire^+^ mTEC, H2‐Aa^−/−^, and Ciita^IV−IV−^ mice lacking MHC class II expression and CD4^+^ CD8^−^ thymocytes have a dramatic reduction in Aire^+^ mTEC 87. Interestingly, TCRα^−/−^ mice deficient in both CD4^+^ CD8^−^ and CD4^−^ CD8^+^ thymocytes appear to have a more severe disruption of the mTEC compartment compared to mice lacking CD4^+^ CD8^−^ thymocytes alone 96, suggesting that other αβTCR‐expressing cell types are capable of inducing mTEC maturation. Interestingly, and in line with this, CD1d^−/−^ mice that lack iNKT cells have a reduction in Aire^+^ mTEC 97. Moreover, during early stages in their intrathymic development, iNKT cells were shown to express both RANKL and CD40L, which decreases as they mature. Combined, these data suggest that in addition to mainstream αβT‐cell development and invariant γδT‐cell development, intrathymic iNKT‐cell development also involves TNF receptor ligand expression, which contributes to the maturation of mTEC 97.

### Lymphotoxinβ receptor (LTβR)

LTβR is expressed by thymic stromal cells and DC but not by developing thymocytes, and two ligands for LTβR have been identified; LIGHT and LTα1β2. Mice deficient in LTβR signaling have disrupted medullary architecture and defects in mTEC populations, including a reduction in terminally differentiated involucrin^+^ mTEC 98. Interestingly, unlike CD40^−/−^ or RANK^−/−^ mice, disruption of LTβR signaling does not alter the numbers of Aire^+^ mTEC. However, mice still show signs of autoimmunity 98, 99, 100, which may be due to alterations in medullary organization rather than altered mTEC^hi^ development. Of note, one study sorted mTEC populations from LTα^−/−^ and LTβ^−/−^ mice, and showed a reduction in both Aire‐dependent and Aire‐independent TRA expression 101, raising the possibility that LTβR may influence mTEC expression of TRA indirectly. Recently, Lkhagvasuren *et al*. 75 devised a method to allow the detection of CCL21 by flow cytometry and showed CCL21 expression predominately within CD80^low^ mTEC. In addition, LTβR^−/−^ mice were shown to have a reduction in CCL21^+^ mTEC 75. Whether this CCL21^+^ mTEC^lo^ population is generated from CCL21^−^ mTEC^lo^ progenitors, or arises later from mTEC^hi^ during post‐Aire stages of development 102, 103, is currently unknown (*Fig.* 1). While the positioning of CCL21^+^ mTEC^lo^ in current models of mTEC development remains to be elucidated, these experiments provide evidence that LTβR plays a direct role in the regulation of the mTEC^lo^ compartment, and may provide an explanation of the importance of LTβR in thymic medulla organization and tolerance induction 99. Most recently, the role of LTβR in thymic tolerance has been examined by Takaba *et al*. 104, who described Fezf2 as a transcription factor that is expressed by mTEC in an LTβR‐dependent manner. Importantly, Fezf2 was shown to control expression of a small range of TRA independently of Aire, supporting the idea that both Aire and Fez2 collectively contribute to tolerance to self‐antigens. Interestingly, while mice deficient in both LTβR ligands (LTβ^−/−^ LIGHT^−/−^) show disrupted medulla formation, the phenotype does not fully recapitulate that seen in the LTβR^−/−^ mice, suggesting that an additional unknown ligand for LTβR might exist 99. Finally, the role of LTβR in co‐operation with other TNFRSF members has been investigated during mTEC differentiation. While mice deficient in both LTβR and CD40 had no additional medullary defects compared to mice deficient in LTβR alone, mice deficient in both LTβR and RANKL showed a greater reduction in mTEC compared to RANKL or LTβR single knockout mice. Moreover, stimulation of FTOC with agonistic anti‐LTβR was reported to induce RANK expression by mTEC, suggesting that initial conditioning via LTβR is important during mTEC development by enabling effective RANK–RANKL‐mediated interactions 91. Whatever the relationship between LTβR and RANK, it is clear that several TNFRSF members combine to ensure normal development, organization, and function of the thymus medulla, and that various cells contribute TNFRSF ligand expression as a basis of thymic crosstalk.

## The thymus medulla and conventional αβT‐cell development

### Cortex to medulla migration

**Figure 2:**
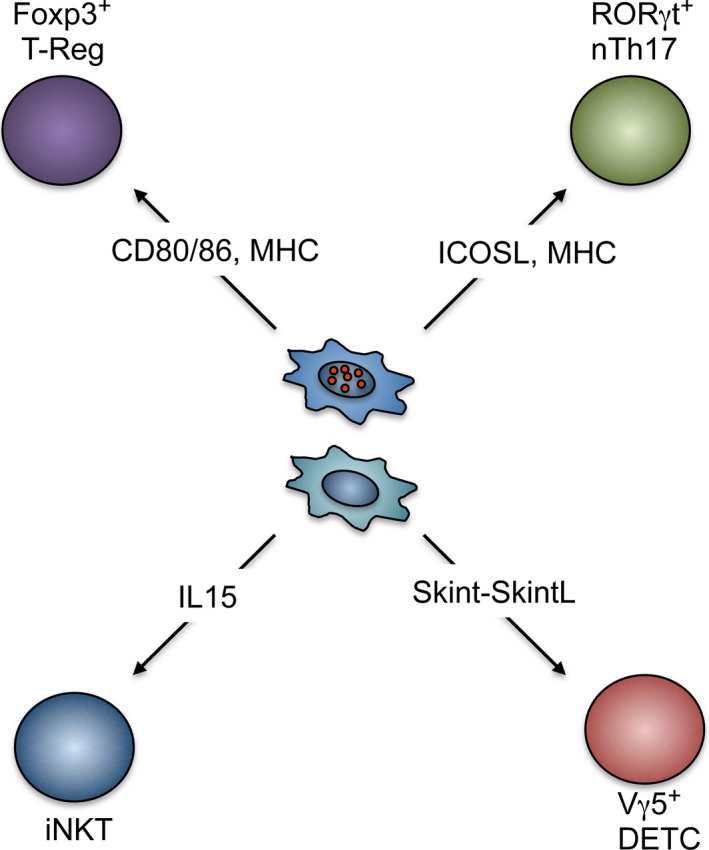
**Regulation of T‐cell development by medullary thymic epithelial cell (m** **TEC** **).** Although the majority of thymocytes that reside within the thymic medulla are of the conventional αβT‐cell lineage, several studies have now revealed the importance of this microenvironment in the generation of other thymus‐dependent T‐cell subsets. However, in most cases, the precise mTEC compartments that regulate development of their development are not fully understood. Of note, these T‐cell sub‐lineages represent components of both the innate and adaptive immune systems, and their development within the thymus has also been shown to be linked to mTEC development, indicating the importance of reciprocal interactions between multiple T‐cell subsets and mTEC progenitors for medulla formation.

Given the critical role of medullary thymic microenvironments in central tolerance enforcement, the migration of thymocytes into the medulla represents a defining step during intrathymic T‐cell development. While the thymic medulla has been known to influence the fate of conventional αβT cells following their positive selection in the cortex, many other studies have also revealed its important role in supporting the development of a range of T‐cell subsets including Foxp3^+^ T‐Reg, CD1d‐restricted iNKT cells, natural Th17 cells, and invariant Vγ5^+^ dendritic epidermal T cells 52, 90, 97, 105, 106. Such events are summarized in *Fig.* 2 and have been recently reviewed elsewhere 107. For conventional αβT cells, the capacity of positively selected thymocytes to enter the medulla is controlled by a fine regulatory balance between cortical retaining and medullary attractant signals. Following positive selection, CD69^+^ thymocytes expressing the chemokine receptor CCR9 are released from cortical retention. At least in part, this release occurs following Semaphorin 3E‐mediated ligation of the transmembrane glycoprotein PlexinD1 expressed by CD4^+^ CD8^+^ thymocytes. Notably, PlexinD1 binding leads to the suppression of CCR9 sensitivity to cTEC‐derived CCL25 108, 109. Consistent with this, absence of PlexinD1 in developing thymocytes leads to the ectopic cortical accumulation of single‐positive thymocytes, highlighting a potential dominant role for CCR9 in the positioning of positively selected thymocytes.

The opposing localization of single‐positive thymocytes within the medulla is positively regulated by CCR7 signaling. In this regard, mice deficient for either CCR7 or the cognate ligands CCL19 and CCL21 demonstrate a failure to accumulate single‐positive thymocytes within the medulla and consequently exhibit defects in negative selection 110, 111, 112. Interestingly however, we 113 and others 114 reported that for CD4^+^ thymocytes, upregulation of CCR7 does not occur immediately following positive selection, but rather takes place at later stages of single‐positive thymocyte maturation. Given that postpositive selection thymocytes exhibit increased directed migration to the medulla 115, it is highly likely that additional positive chemoattractant signals, beyond the release from cortical retention via PlexinD1 activity, regulate attraction of thymocytes to the medulla. In agreement with this, broad inhibition of thymocyte chemokine receptor signaling via pertussis toxin administration in both *in vitro* and *in vivo* systems leads to a reduced capacity of single‐positive thymocytes to accumulate within the medulla 112, 114.

In the search for potential chemokine receptors that regulate guided medullary migration of thymocytes prior to upregulation of CCR7 expression, CCR4 emerged as an interesting candidate. Notably, work by our group and others revealed that CCR4 demonstrated a highly restricted pattern of expression during thymocyte development, exhibiting rapid upregulation by CD69^+^ thymocytes following positive selection 113, 114, 116. Correspondingly, postpositive selection thymocytes demonstrate an *in vitro* capacity to migrate toward the CCR4 ligands CCL17 and CCL22 116 that are expressed by both mTEC 117 and thymic DC 118 within thymic microenvironments. However despite these clues, analysis of CCR4‐deficient mice by our laboratory did not reveal an apparent role for CCR4‐mediated signaling in medullary localization of single‐positive thymocytes, nor thymocyte maturation, including the development of T‐Reg that we previously demonstrated to be medullary‐dependent 52. Moreover, analysis of CCR4/CCR7 double knockout mice revealed that CCR4 appeared to be dispensable even in the absence of CCR7, ruling out potential redundancy between these two chemokine receptors. Interestingly a recent study, using an *ex vivo* thymic slice culture system, revealed that CCR4‐deficient thymocytes exhibit reduced medullary migration of the earliest CD69^+^ postpositive selection thymocytes 118. Moreover, such CCR4‐deficient animals were reported to exhibit impaired thymocyte deletion and further manifested lacrimal gland lymphocytic accumulations and increased autoantibodies, suggestive of a role for CCR4‐mediated migration in T‐cell tolerance. Interestingly, while CCR4 appears to regulate CD69^+^ CD4^+^ CD8^+^ thymocyte medullary entry and interactions between single‐positive CD4^+^ thymocytes and thymic DC, it remains to be precisely ascertained how differential chemokine signaling regulates the balance of DC versus mTEC‐mediated central tolerance, including the differential outcomes of either negative selection or T‐Reg development.

### Single‐positive thymocyte maturation

Following cortical to medullary migration, thymocytes are estimated to reside within medullary microenvironments for a period of approximately 1 week 119, 120, 121. Using two‐photon imaging of *ex vivo* thymic explant cultures, studies have demonstrated that single‐positive thymocytes adopt a ‘random walk’ pattern of movement restricted to limited medullary subzones estimated to be 30 μm in diameter 122. During such medullary residency, thymocytes undergo progressive maturation prior to thymic export and are co‐ordinately subjected to both negative selection events and enforcement of T‐Reg development. Semi‐mature single‐positive thymocytes can be defined by changes in a panel of differentiation markers including expression of CD69, CD24, CD62L, and Qa2. Thus, while newly selected thymocytes have a CD69^+^ CD24^+^ CD62L^−^ Qa2^−^ phenotype, with progressive development their more mature descendants are CD69^−^ CD24^low^ CD62L^+^ Qa2^+^. While semi‐mature single‐positive thymocytes defined by CD24 expression are susceptible to deletion as a result of TCR triggering, more mature CD24^−^ thymocytes respond to the same stimulation via proliferation, suggesting that the maturational status of medullary‐resident thymocytes dictates their susceptibility to negative selection events 123.

The necessity for functional medullary thymic microenvironments to enforce central tolerance is underpinned by experiments documenting aberrant negative selection and T‐Reg development in mice with mTEC defects. Interestingly, in addition to imposing central tolerance, analysis of mice deficient in RelB, where mTEC development is greatly impaired, revealed an absence of the most mature Qa2^+^ CD4^+^ CD8^−^ thymocytes suggesting that mTEC support the progressive maturation of conventional thymocytes 124. However, these studies did not rule out a possible hematopoietic intrinsic role of RelB, or the compounding effect of peripheral autoimmunity that occurs in such mice 77. Using transplantation of RelB‐deficient fetal thymus grafts into WT mice, we found that normal phenotypic progression of CD4^+^ CD8^−^ thymocyte maturation occurred in the absence of RelB‐dependent mTEC. Consistent with this, we found that following intravenous injection, newly selected thymocytes were able to complete their maturation extrathymically, providing support for medullary‐independent maturation of conventional SP4 thymocytes 52. The ability of immature CD4^+^ CD8^−^ thymocytes to mature in an mTEC‐independent manner may be linked to the potential of peripheral DC to support the maturation of RTE 125. Although how these mechanisms operate is still unclear, it may involve interactions of newly produced T cells with secondary lymphoid organ‐resident DC that have been shown to promote phenotypic maturation of thymocytes in *in vitro* co‐culture systems 126. Interestingly, our experiments in which RelB‐deficient thymi were engrafted into WT mice demonstrated the presence of host DC within thymus grafts 52. Combined with *in vitro* studies demonstrating the ability of DC to upregulate Qa2 expression on immature thymocytes 126, this suggests a potential role for thymic DC in the regulated development of CD4^+^ CD8^−^ thymocytes. Given that Aire‐deficient mice are also reported to display defects in CD4^+^ CD8^−^ thymocyte maturation for poorly defined reasons 124 and that DC positioning is defective in Aire‐deficient animals 127, the requirement for thymic DC activity and localization in the regulation of intrathymic thymocyte maturation warrants investigation.

### Thymocyte egress

Following intrathymic selection events, single‐positive thymocytes acquire the ability to egress from thymic microenvironments. Although it was previously unclear whether the exit from the thymus occurred in a random fashion independent of maturational status 128, recent studies have indicated that this process may involve a strictly regulated mechanism. Notably, only mature single‐positive thymocytes upregulate transcriptional regulators of thymic egress including Foxo1 and Klf2, which control expression of the sphingosine‐1 phosphate‐1 receptor (S1P_1_). Expression of the G‐protein‐coupled receptor S1P_1_ confers the ability of thymocytes to migrate toward high concentrations of the ligand S1P. Critically, the concentration of S1P is highest within the blood and within thymic microenvironments; neural crest‐derived pericytes lying in close physical approximation to blood vessels at the thymic corticomedullary junction play key roles in the production of active S1P 129. The significant role of S1P:S1P_1_ interactions in the regulation of thymic egress via reverse transendothelial migration has been highlighted by several studies demonstrating that abrogation of S1P gradients or S1P_1_‐mediated signaling leads to a blockade in mature SP thymocyte egress and their concomitant retention in thymic perivascular sheathes 130, 131, 132, 133.

In addition to the role of S1P in the emigration from thymic microenvironments, mice deficient for expression of LTβR have been reported to exhibit retention of mature CD62L^+^ thymocytes within the thymus 99. Although the precise mechanism of LTβR‐mediated regulation of thymocyte egress is unknown, interestingly LTβR‐deficient thymi have not been reported to exhibit overt perivascular accumulations that occur as a result of dysregulated S1P signaling, perhaps suggesting that LTβR regulates alternative pathways controlling thymic egress. Notably, the chemokines CCL19 and CCL21 represent known LTβR targets 134. Although CCL21 has not been implicated in thymic egress events, disruption of CCL19 signaling via *in vivo* antibody‐mediated neutralization leads to a reduction in thymic emigration at least during neonatal stages 135.

## The thymus medulla controls Foxp3^+^ T‐cell development

In addition to the clonal deletion of autoreactive T cells in the medulla, the thymus actively maintains T‐cell tolerance through the generation of natural T‐Reg. The T‐Reg lineage is defined by the forkhead box family transcription factor Foxp3, the expression of which is both necessary and sufficient for T‐Reg to mediate antigen‐specific suppression of peripheral T‐cell responses 136. Mice deficient in T‐Reg through natural mutation of the X‐linked *Foxp3* gene – ‘scurfy’ mice – develop fatal autoimmune disease a few weeks postpartum 137, demonstrating the absolute requirement of T‐Reg in maintaining immune tolerance in the periphery. Emergence of the T‐Reg lineage in the thymus occurs at an intermediate point in the development of CD4^+^ T cells in the medulla that can be defined by cell surface expression of CD69 and the chemokine receptor CCR7 52. T‐Reg development has a particular set of requirements relative to the intrathymic generation of conventional CD4^+^ T cells, including appropriate levels of TCR triggering 138, co‐stimulation, and γ‐chain cytokine signaling 139, 140, 141, 142. Foxp3^+^ T‐Reg are only detectable postpartum in the murine thymus 143, coinciding with the maturation of the medulla, where distinct stromal populations in mTEC and DC provide a unique niche for T‐Reg selection. Recently we showed an absolute requirement for mTEC in the induction of T‐Reg development 52. As development of the mTEC lineage is blocked in the absence of Relb expression 78, 79, by grafting wildtype or Relb^−/−^ fetal lobes under the kidney capsule of wildtype mice we were able to assess the necessity for mTEC in the development of conventional T cells and T‐Reg. In Relb^−/−^ lobes, we observed almost complete abrogation of T‐Reg generation, including their CD25^+^ Foxp3^−^ precursors 52. In contrast, the development and maturation of conventional CD4^+^ CD8^−^ thymocytes proceeded normally in mTEC‐deficient lobes, including the generation of mature Qa2^hi^ CD69^−^ cells 52. Such findings are of interest as they indicate a differential requirement for mTEC in conventional and T‐Reg development that underscores the importance of the thymic medulla for T‐cell tolerance induction.

In Nur77‐GFP mice, in which levels of GFP in thymocytes are an indicator of the strength of TCR triggering, T‐Reg have a higher level of GFP compared to conventional CD4^+^ CD8^−^ thymocytes 138, suggesting that they undergo high‐affinity antigen recognition during their development. Indeed, through mixed bone marrow chimeras including an increasingly dilute fraction of G113 TCR‐transgenic cells, antigen‐specific T‐Reg were shown to compete for their selecting ligand. Higher fractions of Foxp3^+^ cells were detectable among transgenic T cells when they represented only a small proportion of thymocytes 138, and hence were better positioned to receive TCR stimulation above the affinity required for Foxp3 induction. Through expression of the transcription factor Aire, and the regulation of self‐antigen expression, mTEC have an effective capacity to provide high‐affinity TCR interactions leading to T‐Reg generation. Interestingly, while this fits with the observation that T‐Reg are reduced in the Aire^−/−^ thymus, this is likely complicated by the finding that DC positioning within the medulla is controlled by Aire‐dependent chemokine expression 127. However, studies on the generation of antigen‐specific T‐Reg show that cells with TCR specificities to known Aire‐dependent self‐antigens fail to develop in the absence of Aire expression 144, 145, 146 providing strong evidence that Aire plays a direct role during T‐Reg thymic development.

Recently, T‐Reg generation was viewed as a defined two‐step process, wherein Foxp3^−^ precursors formed through strong TCR engagement upregulate the high‐affinity IL2Rα chain CD25, and gain responsiveness to thymic IL‐2, allowing subsequent expression of Foxp3 142. This precursor population is again identifiable by high levels of Nur77‐GFP 138, and like mature T‐Reg, CD25^+^ Foxp3^−^ cells are greatly reduced in Relb^−/−^ thymus grafts 52, suggesting a reliance on high‐affinity antigen recognition in the initial stages of T‐Reg lineage commitment. Moreover, interactions between thymocytes and mTEC/DC involving TNFRSF members and their ligands have been reported to be required for T‐Reg development. It is known that CD80/86 co‐stimulation is required for the induction of CD25^+^ precursors 139, 142, 147 and TCR signaling and CD80/86 co‐stimulation together are required for subsequent upregulation of a cluster of other TNFRSF members on the surface of T‐Reg precursors, including OX40, GITR, and TNFR2 141. Interestingly, the corresponding ligands are expressed by both mTEC and DC within the thymic medulla, and provision of co‐stimulation through these receptors appears to be required for the generation of CD25^+^ precursors, and their efficient conversion into Foxp3^+^ T‐Reg 141. Similarly, stimulation via CD27, a TNFRSF member expressed by CD4^+^ CD8^−^ thymocytes, by medullary APC‐derived CD70 has been shown to be important in thymic T‐Reg generation 140. However, as CD27 expression does not appear to be a direct indicator of TCR signaling strength, and T‐Reg survival is altered in CD27^−/−^ mice 140, CD27–CD70 interactions may be part of a distinct but complimentary developmental pathway during intrathymic T‐Reg generation.

A more recent view of T‐Reg development and its control was provided by Tai *et al*. 148, who demonstrated the existence of T‐Reg precursors with a Foxp3^+^ CD25^−^ phenotype. This population was shown to have a unique challenge in their development, as Foxp3 conferred a pro‐apoptotic signature to cells, typified by accumulation of PUMA and active Bim, both regulators of apoptosis. Rescue from cell death during these early stages of T‐Reg development was shown to be provided by IL‐2 signaling after upregulation of CD25, which triggers downstream expression of the pro‐survival molecule Bcl‐2 148. As this discovery of distinct subsets of T‐Reg precursors is followed by further analysis of their developmental requirements, a clearer picture of the mechanisms that control Foxp3^+^ T‐Reg development in the thymus should emerge.

It is clear from several studies that mTEC are important regulators at multiple stages of T‐Reg differentiation. When combined with the increasing understanding of signaling pathways involved in mTEC homeostasis, the thymic stroma has become an increasingly appealing target for drugs aiming to alter the balance of production of conventional T cells and T‐Reg. Indeed, Khan *et al*. 92 demonstrated that through treatment of mice with blocking anti‐RANK antibodies, the homeostasis of Aire^+^ mTEC could be disrupted which limited the development of Foxp3^+^ T‐Reg and allowed the escape of autoreactive T cells from negative selection. Interestingly, this escape from central tolerance mechanisms was sufficient to increase the efficiency of anti‐tumor immune responses, and prolonged the survival of mice inoculated with B16 melanoma. Further reciprocal studies also focused on how expansion of the mTEC compartment might be a means to boost production of T‐Reg. Mice in which Aire^+^ mTEC are expanded as a result of disruption of homeostatic pathways, including those lacking either TEC expression of TGFβRII 149 or OPG 150, have been used as models to investigate this. Interestingly, numbers of thymic T‐Reg are increased in both models. Moreover, ablation of T‐Reg in mice lacking TEC expression of TGFβRII displayed a mild autoimmune phenotype 149, while enhanced tumor growth was noted in nude mice co‐transplanted with OPG‐deficient fetal thymus 150, suggesting that peripheral tolerance could be enhanced in the absence of negative regulation of mTEC development.

These findings are supportive of the idea that the development of T‐Reg is limited by the availability of mTEC in the normal thymus. However, through use of RAG2pGFP mice, in which developing thymocytes are transiently induced to express GFP, thymic T‐Reg have been found to be a heterogeneous population consisting of both newly produced GFP^+^ cells and GFP^−^ cells that represent recirculating mature peripheral T cells 119. In light of this, we investigated whether the increase in thymic T‐Reg seen in models with expanded mTEC compartments mapped to *de novo* Rag2pGFP^+^ versus recirculating Rag2GFP^−^ T‐Reg in the thymus. We crossed OPG‐deficient mice with RAG2pGFP mice, and found that while thymic T‐Reg numbers were indeed increased, this could be explained by an increase specifically in the recirculating Rag2pGFP^−^ fraction 72. Moreover, as T‐Reg recent thymic emigrants were present in normal numbers in these mice 72, our data argue against the idea that increasing the mTEC population size enhances the efficiency of T‐Reg generation. Interestingly, while two recent publications have demonstrated the capacity of recirculating T‐Reg to actively constrain *de novo* thymic T‐Reg production by competing for DC‐derived IL‐2 151, 152, enhanced thymic T‐Reg recirculation seen in OPG^−/−^ mice did not further reduce T‐Reg development. Further studies are required to determine the control of peripheral T‐Reg recirculation back to the thymus, and its impact on intrathymic T‐cell development. Collectively, such studies show that T‐Reg development involves a complex series of events that controls their optimal intrathymic production. Current findings suggest considerable potential in the exploitation of pathways regulating mTEC, and their provision of co‐stimulation, in tailoring the output of T‐Reg for treatment of autoimmune disease and cancer.

## Concluding remarks

The intrathymic generation of self‐tolerant CD4^+^ CD8^−^ and CD4^−^ CD8^+^ αβT cells requires controlled migration through the thymus. The thymic medulla plays a key role in this process by mediating the negative selection of thymocytes with the potential to generate autoimmune responses to self‐antigens. Additionally, the medulla plays an important role in maintaining tolerance in peripheral tissues by supporting the generation of Foxp3^+^ T‐Reg. Critical to medulla function is the establishment of mTEC environments that influence multiple aspects of intrathymic T‐cell development. Significant progress has been made in understanding the pathways that control mTEC development, including identification of mTEC stem cells, and the role of TNFRSF molecules. However, our knowledge of mTEC development remains incomplete, and further studies are required to understand the formation and maintenance of mTEC microenvironments, particularly in the steady‐state adult thymus and following thymic injury. Additionally, how mTEC are able to control the balance between negative selection and the production of conventional and Foxp3^+^ T‐Reg is poorly understood, and will require further detailed investigation of mTEC heterogeneity at both the phenotypic and functional level. Ultimately, gaining a clearer understanding of the control of the thymic medulla will aid in understanding and manipulating self/non‐self discrimination that determines the balance between tolerance and immunity.
